# *Talaromyces marneffei* and nontuberculous mycobacteria co-infection in HIV-negative patients

**DOI:** 10.1038/s41598-021-95686-0

**Published:** 2021-08-10

**Authors:** Ye Qiu, Jie Huang, Yu Li, Wen Zeng, Mianluan Pan, Jiemei Cen, Hui Zhang, Xuejiao Sun, Dongming Qu, Jianquan Zhang

**Affiliations:** 1 Department of Respiratory and Critical Medicine, The Eighth Affiliated Hospital, Sun Yat-Sen University, Shenzhen, 518000 Guangdong China; 2 Department of Comprehensive Internal Medicine, The Affiliated Tumor Hospital of Guangxi Medical University, Nanning, 530021 Guangxi China; 3 Department of Respiratory and Critical Medicine, The First Affiliated Hospital of Guangxi Medical University, Nanning, 530021 Guangxi China; 4 Department of Tuberculosis Ward, Nanning Forth People’s Hospital, Nanning, 530021 Guangxi China; 5 Department of Respiratory and Critical Medicine, Yiyang Central Hospital, Yiyang, 413000 Hunan China; 6 Department of Respiratory and Critical Medicine, Liuzhou First People’s Hospital, Liuzhou, 545000 Guangxi China; 7 Department of Respiratory and Critical Medicine, Nan Xishan Hospital of Guangxi Zhuang Autonomous Region, Guilin, 541000 Guangxi China

**Keywords:** Immunology, Microbiology, Bacteria, Clinical microbiology, Fungi, Infectious-disease diagnostics

## Abstract

To describe the clinical features and the risk factors for nontuberculous mycobacteria (NTM) and *Talaromyces marneffei* (TM) co-infections in HIV-negative patients. A multicenter retrospective study in 13 hospitals, and a systematic literature review were performed of original articles published in English related to TM/NTM co-infections. HIV-negative patients with TM and NTM co-infections comprised Group 1; TM-only infection Group 2; NTM-only infection Group 3; and healthy volunteers Group 4. Univariate logistic analysis was used to estimate the potential risk factors of TM/NTM co-infections. A total of 22 cases of TM and NTM co-infections were enrolled. Of these, 17 patients (77.3%) had a missed diagnosis of one of the TM or NTM pathogens. The anti-IFN-γ autoantibodies (AIGAs) titer, white blood cell (WBC), neutrophil counts (N), erythrocyte sedimentation rate (ESR), C reactive protein (CRP), globulin, and immunoglobulin G (IgG) levels of Group 1 were higher than those of the other groups, whereas the levels of CD4^+^T cells was lower than those of other groups. There was a significant negative correlation between the AIGA titers and the number of CD4^+^T cells (*P* < 0.05). Factors including the ratio of the actual values to the cut-off values of AIGAs, WBC, N, HGB, CD4^+^T cells, IgG, IgM, IgA, serum globulin, ESR, and CRP were taken as potential risk factors for TM and NTM co-infection. Most patients with TM and NTM co-infection had a missed diagnosis of one of the TM or NTM pathogens. The levels of AIGAs, WBC, N, ESR, and CRP in TM and NTM co-infections were remarkably higher than in mono-infection. High-titer AIGAs may be a potential risk factor and susceptibility factor for co-infection of TM and NTM in HIV-negative hosts.

*Talaromyces marneffei* (TM) and nontuberculous mycobacteria (NTM) are opportunistic intracellular pathogens, with a strong association toward acquired immunodeficiency syndrome (AIDS) and other immunocompromised conditions^1–4^. Recently, an increasing number of TM and NTM mono-infection have been reported in HIV-negative patients^5–7^, especially in adults producing anti-IFN-γ autoantibodies (AIGAs)^8–12^. Refractory and relapsing TM and NTM infections often occur due to the high rates of misdiagnosis and inappropriate therapy leading to poor prognosis^13,14^. Thus, timely diagnosis of TM and NTM co-infections and differential diagnosis between mono- and co-infections are key to improve prognosis.

However, systematic clinical cohort studies of TM and NTM co-infections in HIV-negative hosts are lacking. Here, we report 22 HIV-negative adult patients, who suffered from co-infections by TM and NTM due to AIGAs. This study aimed to describe the clinical features and address the risk factors for NTM and TM co-infections in HIV-negative individuals.

## Results

### Patient demographics

A total of 22 HIV-negative patients with disseminated TM and NTM co-infection were enrolled in our study Group 1, including 14 patients from the multicenter retrospective cohort and 8 patients from the literature review cohort^13–19^.

In Group 1, simultaneous diagnosis with TM and NTM co-infections (Group 1A) was only made in 5 patients. The majority of patients with TM/NTM co-infections (17 patients, 77.3%) was firstly diagnosed with only one of the pathogens (group 1B), including 8 cases of initially missed diagnosis of TM, and 9 cases of initially missed diagnosis of NTM. TB was the most common presumed diagnosis in groups 1, 2 and 3. TB was the most common misdiagnosis in Groups 1, 2, and 3. Baseline patient characteristics are presented in Table 1. Sex, age, and underlying disease distribution were not significantly different between the three groups.

**Table 1:** Baseline demographics and clinical characteristics of the 106 participants.

Variable	Group 1 (n = 22)	Group 2 (n = 22)	Group 3 (n = 22)	Group 4 (n = 40)	*P-*value
Age (year)	52 (42, 57)	61 (46, 66)	60 (50, 62)	49 (33, 57)	0.180
Sex, female n (%)	9 (40.9)	8 (36.4)	11 (50.0%)	22 (55.0)	0.480
BMI (kg/m^2^)	19.5 (18.2, 20.4)	19.5 (17.4, 22.6)	19.5 (17.0, 21.6)	–	0.915
Underlying disease*	9 (40.9)	9 (40.9)	7 (31.8)	–	0.798
AIGAs positive**	20 (100.0)^a, b, c^	12 (54.5)^d^	8 (36.4)	0 (0)	**0.000**
AIGAs titers (ng/mL)^g^	58,931.1 (32,343.8, 81,530.2)^a, b, c^	16,070.4 (3496.1, 24,673.5)^d, e^	12,302.2 (2523.1, 9068.4)^f^	1497.4 (1192.3, 3177.7)	**0.000**
WBC × 10^9^cells/L^g^	21.9 (18.1, 23.9)^b^	20.8 (13.8, 30.3)^d^	7.0 (5.4, 8.4)	ND	**0.000**
N × 10^9^ cells/L^g^	18.5 (13.6, 19.9)^b^	16.3 (11.8, 25.1)^d^	4.5 (3.5, 6.6)	ND	**0.000**
L × 10^9^ cells/L^g^	1.3 (0.8, 1.7)	1.1 (0.62, 2.1)	1.2 (0.9, 1.4)	ND	0.769
HGB g/L^g^	84.0 (60.4, 88.8)^b^	71 (63.0, 97.6)^d^	120 (110.9, 134.8)	ND	**0.000**
ESR mm/h^g^	106.0 (90.0, 119.0)^b^	95.5 (59.6, 113.25)^d^	26.0 (8.0, 49.0)	ND	**0.000**
CRP mg/L^g^	166.9 (136.9, 200.0)^b^	133.6 (92.5, 192.0)^d^	10 (8.9, 13.9)	ND	**0.000**
CD4^+^T cell cells/μL^g^	173 (105, 396)^a, b^	676 (519, 1088)	674 (547, 839)	ND	**0.001**
CD8^+^T cell cells/μL^g^	378 (231, 709.5)	470 (311,852)	378 (231, 709)	ND	0.651
CD3^+^T cell cells/μL^g^	549 (268, 806)^a, b^	1246 (806,1796.7)	1053 (725, 1602.5)	ND	**0.013**
IgG g/L^g^	29.5 (18.8, 39.1)^a, b^	22.5 (12.3, 28.0)^d^	14.3 (10.1, 18.2)	ND	**0.003**
IgA g/L^g^	2.3 (2.1, 4.3)	2.7 (2.3, 3.5)	2.4 (1.7, 4.0)	ND	0.931
IgM g/L^g^	2.0 (1.2, 2.9)^a, b^	1.1 (0.6, 1.8)	0.71 (0.7,1.6)	ND	**0.004**
Globulin g/L^g^	45.9 (36.8, 53.55)^a, b^	33.7 (21.7. 58.9)^d^	28.5 (24.2, 37.9)	ND	**0.011**

Bold values indicate significant difference between groups or in univariate logistic regression analysis.

^a^Indicates statistical significance between Groups 1 and 2.

^b^Indicates statistical significance between Groups 1 and 3.

^c^Indicates statistical significance between Groups 1 and 4.

^d^Indicates statistical significance between Groups 2 and 3.

^e^Indicates statistical significance between Groups 2 and 4.

^f^Indicates statistical significance between Groups 3 and 4.

^g^A total number of 14 patients in Group 1 had AIGAs titer, WBC, N, L, HGB, ESR, CRP, globulin, immunoglobulins (IgG, IgA, IgM), and lymphocytes subpopulations (CD4^+^T cell, CD8^+^T cell, CD3^+^T cell) data.

Data are expressed as median ± interquartile range. Kruskal–Wallis H test was used to determine statistical significance among the 3 or 4 groups, followed by a 2 by 2 comparison across groups through a Fisher’s exact test. *P* < 0.05 indicates statistical significance.

Group 1 = patients with TM and NTM co-infections; Group 2 = patients with TM mono-infection; Group 3 = patients with NTM mono-infection; Group 4 = healthy control volunteers.

*Indicates the nature of the underlying disease in three groups. Group 1: 5 cases with Sweet’s syndrome, 1 case with malignant tumor, 1 case with cystic fibrosis, 1 case with Behcet’s syndrome, and 1 case with diabetes; Group 2: 1 case with thalassemia, 1 case with Sjogren's syndrome, 1 case with ankylosing spondylitis, 1 case with major trauma or surgery, 1 case with hyperthyroidism, 2 cases with glucocorticoids and or immunosuppressive agents, 1 case with hypertension, and 1 case of diabetes. Group 3: 3 cases with major trauma or surgery, 3 cases with hypertension, and 1 case with diabetes.

**Serums from 14 participants in Group 1, all patients in Groups 2 and 3, and 40 health volunteers were tested for anti-IFN-γ autoantibodies. Six of eight patients in the literature review cohort in Group 1 were defined as AIGA-positive, while the last 2 patients were not assessed. Thus, a total of 20 patients were tested for AIGAs in Group 1.

*BMI* body mass index, *AIGAs* anti-IFN-γ auto-antibodies, *ND* no data, *WBC* white blood cell, *N* neutrophil counts, *L* lymphocyte counts, *HGB* haemoglobin, *ESR* erythrocyte sedimentation rate, *CRP* C-reactive protein, *Ig* immunoglobulin. Normal range: IgG, 8–18 g/L; IgA, 2.01–2.69 g/L; IgM, 0.84–1.32 g/L; CD4^+^T cell, 410–1590 cells/μL; CD8^+^T cell, 190–1140 cells/μL; CD3^+^T cell, 690–2540 cells/μL.

### Laboratory findings and clinical features

**Figure 1:**
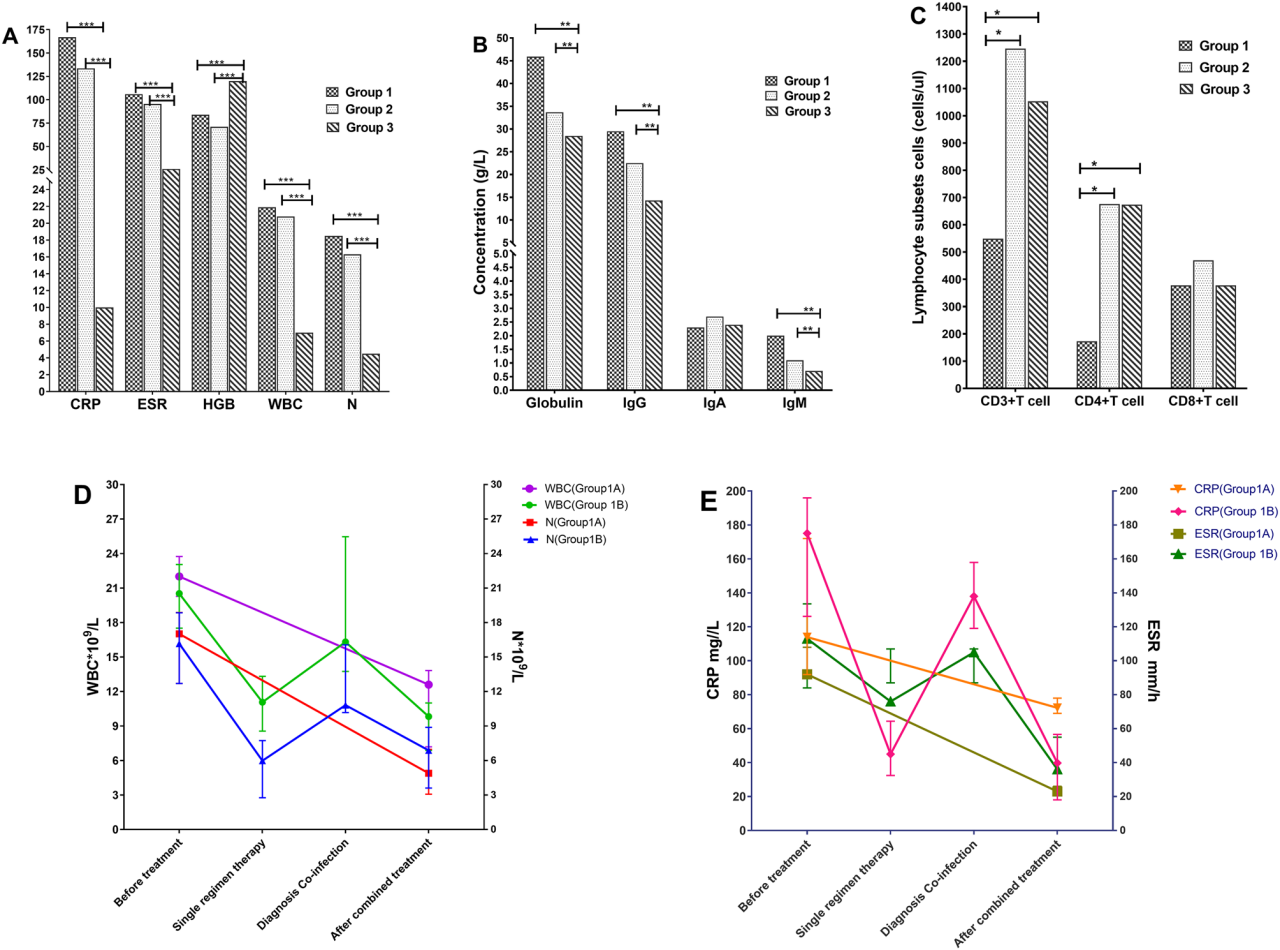
Comparison of biochemical indexes between groups. (**A**) White blood cell (WBC), neutrophil (N) counts, erythrocyte sedimentation rate (ESR), and C reactive protein (CRP) were significantly increased in Groups 1 and 2 compared with Group 3 (*P* < 0.001). (**B**) Globulin, IgG, and IgM in Group 1 were higher than normal reference values and higher than Groups 2 and 3 (*P* < 0.01). (**C**) CD4^+^T and CD3^+^T-lymphocyte counts in Group 1 were lower than normal reference values and lower than Groups 2, 3, and 4 (*P* < 0.01). (**D**, **E**) After combined treatment, all parameters in patients were improved.

Laboratory findings are shown in Table 1 and Fig. 1. Routine bloodwork including, erythrocyte sedimentation rate (ESR), C reactive protein (CRP) lymphocyte phenotyping, and serum immunoglobulin G (IgG)] were performed for 14 patients from the retrospective study, and were not available in patients from the literature review cohort. White blood cell (WBC), neutrophil counts (N), ESR, and CRP in Groups 1 and 2 were significantly higher than in Group 3 (*P* < 0.001). Hemoglobin (HGB) in Groups 1 and 2 were lower than in Group 3 (*P* < 0.05). Globulin, IgG, and IgM levels of Group 1 were higher than those of the other groups. CD4^+^ T and CD3^+^ T lymphocyte counts in Group 1 were lower than normal reference values and that in Groups 2 and 3, respectively (*P* < 0.05) (Table 1, Fig. 1A–C).

Significant differences in clinical manifestations were found (*P* = 0.002) through Chi-square statistical tests for all the clinical manifestations between Groups 1, 2, and 3 in the three groups (Table 2). The most common clinical features in Group 1 were lymphadenopathy, fever, and cutaneous lesions, followed by cough, weight loss, and ostealgia. Fever, lymphadenopathy, and ostealgia were more common in Groups 1 and 2. However, weight loss and cough were more common in Group 3. Chest high resolution computed tomography (HRCT) was also conducted in the three groups (Table 2), showing significant differences in the prevalence of mediastinal lymphadenopathy, fibrous cords, pleural effusion and/or pleural thickening, bronchiectasis, and cavitary lesions. Mediastinal lymphadenopathy was more common in Groups 1 and 2, whereas fibrous cord, cavitary lesions, and bronchiectasis were more common in Group 3.

**Table 2:** Symptoms and imaging findings of Chest HRCT in three groups.

Variable	Group 1 (n = 22)	Group 2 (n = 22)	Group 3 (n = 22)	*P*-value
**Symptoms, n (%)**				**0.002**
Fever	19 (86.4)^b^	21 (95.5)^c^	7 (31.8)	**0.000**
Lymphadenopathy	20 (90.9)^b^	20(86.4)^c^	7 (31.8)	**0.000**
Cutaneous lesions	19 (86.4)^a, b^	10 (45.5)^c^	2 (9.1)	**0.000**
Ostealgia	12 (54.5)^b^	11 (50)^c^	1 (4.5)	**0.000**
Weight loss	13 (59.1)^a^	8 (36.4)^c^	16 (72.7)	**0.047**
Cough and sputum production	14 (63.6)^b^	15 (68.2)^c^	22 (100)	**0.007**
Hepatosplenomegaly	4 (18.2)	6 (27.2)	2 (9.1)	0.438
Shiver	4 (18.2)	3 (13.6)	2 (9.1)	0.715
Pectoralgia	8 (36.4)	2 (9.1)	7 (31.8)	0.086
Shortness of breath	5 (22.7)	5 (22.7)	9 (40.9)	0.307
Abdominal pain	3 (13.6)	3 (13.6)	1 (4.5)	0.483
Imaging features of Chest HRCT, n (%)*				**0.023**
**Number of patients assessed**	20	22	22	–
Pulmonary consolidation	17 (85.0)	21 (95.5)	19 (86.4)	0.189
Mediastinal lymphadenopathy	11 (55.0)^b^	10 (45.5)^c^	1 (4.5)	**0.002**
Fibrous cords	8 (40.0)^b^	9 (40.9)^c^	16 (72.7)	**0.032**
Pleural effusion/pleural thickening	12 (60)^b^	18 (81.8)^c^	7 (31.8)	**0.003**
**Nodular lesions**	9 (45.0)	9 (40.9)	4 (18.2)	0.182
Tracheal inflated sign	3 (15.0)	5 (22.7)	1 (4.5)	0.383
Ground glass opacities	3 (15.0)	1 (4.5)	2 (9.1)	0.603
Pericardial effusion	2 (10.0)	7 (31.8)	2 (9.1)	1.000
Bronchiectasis	1 (5.0)^b^	1 (4.5)^c^	7 (31.8)	**0.009**
Cavitary lesions	1 (5.0)^b^	3 (13.6)^c^	9 (40.9)	**0.003**

Bold values indicate significant difference between groups or in univariate logistic regression analysis.

^a^Indicates statistical significance between Groups 1 and 2.

^b^Indicates statistical significance between Groups 1 and 3.

^c^Indicates statistical significance between Groups 2 and 3.

Data are presented as n (%). Fisher’s exact test and Kruskal–Wallis H test were used to calculate *P*-values. *P* < 0.05.

Group 1 = patients with TM and NTM co-infections, Group 2 = patients with TM infections only, Group 3 = patients with NTM infections only. *HRCT* high resolution computed tomography.

*Two patients from the systematic literature review did not undergo HRCT. Thus, a total of 20 patients received HRCT.

These indexes were also compared between Groups 1A and 1B, which showed no significant differences (Supplementary Table 1). However, the CD4^+^T lymphocytes and CD3^+^T lymphocytes in Group 1A were lower than in Group 1B. In addition, patients in Group 1A receiving combined treatment (anti-fungal with anti-NTM treatment) showed a significant decrease in the inflammatory indexes (WBC, N, ESR, and CRP). By contrast, in Group 1B, the inflammatory indexes did not decrease, but rather increased following a single regimen therapy (anti-fungal or anti-NTM treatment). However, upon identifying the second pathogen and providing combined treatment, these inflammatory indexes, symptoms, and signs in patients improved (Fig. 1D, E, Supplementary Table 2).

**Figure 2:**
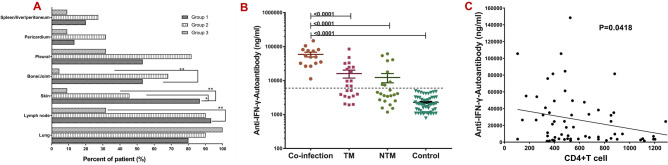
Comparison of sites involved among three groups. (**A**) Lymph nodes were most commonly involved (90.1%), followed by the skin (86.4%) and bone/joint (54.5%) in Groups 1 and 2. (**B**) Comparing the anti-IFN-γ autoantibodies titer between groups, titers in Groups 1, 2, and 3 were remarkably higher than the healthy volunteer group, with patients in Group 1 showing the highest anti-IFN-γ autoantibodies titer. (**C**) The correlation between AIGA titers and the number of CD4^+^T cells.

Comparing the involved sites of the three groups (Supplementary Table 3, Fig. 2A), lymph nodes, skin, and bone/joints were the most commonly infected sites in Groups 1 and 2. Lung involvement was more common in Groups 2 and 3, with pleural as the most commonly involved site in Group 2.

### Microbiology and pathology in patients with concomitant or sequential infections by TM and NTM

TM was most commonly isolated from respiratory specimens (14 cases), including bronchoalveolar lavage fluid (BALF) (7 cases), sputum (5 cases), and lung tissue (2 cases), followed by blood (5 cases), purulent secretion (4 cases), and lymph nodes (3 cases) in Group 1. By contrast, NTM was most commonly isolated from lymph nodes (7 cases), sputum (4 cases), and blood (4 cases) in Group 1.

Granulomatous lesions (12 cases), followed by non-specific inflammation (11 cases) and suppurative lesions (7 cases), were the most common histological findings in 27 pathological specimens from Group 1. Further, positive PAS staining (40.7%) of tissues and secretions were more frequent than acid-fast (AFB) staining (14.8%) in Group 1.

The distribution of rapid- and slow-growing nontuberculous mycobacterial species was similar in Group 1 (Supplementary Table 4). In these patients, the most commonly isolated species was *Mycobacterium abscessus* (4/11, 36.4%), followed by *Mycobacterium chelonae* and *Mycobacterium kansasii* (3/11, 27.6%). In addition to TM and NTM, other common co-infecting pathogens in Group 1 were *Staphylococcus aureus, Aspergillus, Salmonella,* and *Burkholderia*. Moreover, one patient was infected by up to six pathogens during the course of disease.

### Increased AIGA levels in TM and NTM co-infection

Serums obtained from 14 participants in Group 1, all patients in Groups 2 and 3 (n = 22 in each group), and 40 health volunteers were tested for AIGAs. Furthermore, six of the eight patients in the literature review cohort in Group 1 were defined as AIGA-positive, the other 2 patients were not assessed. The positivity rate of AIGAs was significantly different across groups, specifically 100% (20/20), 81.8%, and 63.6% in Groups 1, 2, and 3, respectively (*P* = 0.000). When comparing AIGA titers between the groups, Groups 1, 2, and 3 were remarkably higher than the healthy volunteer group, with Group 1 showing the highest AIGA titer (Table 1, Fig. 2B). Meanwhile, there was a significant negative correlation between AIGA titers and the number of CD4^+^T cells (*P* < 0.05, Fig. 2C).

### Univariate analysis logistic regression analyses for risk factors of TM and NTM co-infections

We analyzed risk factors for developing TM/NTM coinfections in group 1 compared to groups 2 and 3. We found that factors including the ratio of the actual values to the cut-off values of AIGAs, WBC, N, HGB, CD4^+^T cells, IgG, IgM, IgA, serum globulin, ESR, and CRP were taken as potential risk factors for TM and NTM co-infection (Table 3).

**Table 3:** Results of univariate analysis for risk factors of TM and NTM co-infection (n = 66).

Variable	Univariate analysis		
	*P*	HR	95%CI
Age (year)	0.103	0.961	0.917–1.008
BMI (kg/m^2^)	0.948	1.006	0.834–1.214
Relative nAIGA titer	**0.000**	1.840	1.331–2.544
WBC × 10^9^cells/L	**0.018**	1.081	1.013–1.152
L × 10^9^cells/L	0.760	0.998	0.986–1.011
N × 10^9^cells/L	**0.025**	1.083	1.010–1.161
HGB g/L	**0.037**	0.976	0.953–0.998
CD4^+^T cell cells/μL	**0.001**	0.998	0.996–1.000
CD8^+^T cell cells/μL	0.823	1.000	0.999–1.002
CD3^+^T cell cells/μL	0.316	1.000	0.999–1.000
IgG g/L	**0.008**	1.107	1.1027–1.193
IgA g/L	0.856	1.1051	0.616–1.793
IgM g/L	**0.007**	3.892	1.459–10.382
Globulin g/L	**0.020**	1.058	1.009–1.109
ESR mm/h	**0.003**	1.033	1.012–1.056
CRP mg/L	**0.002**	1.019	1.007–1.031

Bold values indicate significant difference between groups or in univariate logistic regression analysis.

Relative nAIGA titer indicates the ratio of the actual value to the cut-off value of nAIGA. *BMI* body mass index, *nAIGAs* neutralizing anti-IFN-γ auto-antibodies, *ND* no data, *WBC* white blood cell, *N* neutrophil counts, *L* lymphocyte counts, *HGB* haemoglobin, *ESR* erythrocyte sedimentation rate, *CRP* C-reactive protein, *Ig* immunoglobulin.

### Treatment and outcome

The prognosis and outcomes of patients in Group 1 was worse than that of patients in Groups 2 and 3, especially in cases of persistent and/or relapsed infections (*P* < 0.001) (Table 4).

**Table 4:** Comparison of the outcomes between three groups in 66 HIV-negative participants.

Variable	Group 1 (n = 22)	Group 2 (n = 22)	Group 3 (n = 22)	*P*-value
**Prognosis and outcomes**				0.043
Cured	7 (31.8)	14 (63.6)	14 (63.4)	
Persistent or relapse infection*	13 (59.1)	2 (9.1)	5 (22.7)	
Death	1 (4.5)	6 (27.3)	2 (9.1)	
Lost	1 (4.5)	0	0	

Data are expressed as number and percentage (%). Fisher’s exact test and Kruskal–Wallis H test were used to determine statistical significance among the groups. *P* < 0.05 was taken as significant.

Group 1 = patients with TM and NTM co-infections, Group 2 = patients with TM infection only, and Group 3 = patients with NTM infection only.

*Persistent or Relapse infection: In Group 1, the infection condition of patients may have been only TM persistent infection, only TM recurrent infection, only NTM persistent infection, only NTM recurrent infection, both TM persistent and recurrent infection, both NTM persistent and recurrent infection, both TM persistent infection and NTM recurrent infection, or both NTM persistent infection and TM recurrent infection. Detailed prognostic information for Group 1 can be found in Table 3 which described the treatment and patient outcomes in Group 1. In Group 2, there was one case with persistent infection and one case with relapse infection. In Group 3, there was one case with persistent infection and four cases with relapse infections.

Treatment outcomes are presented in Table 3 among 22 patients: 19 received anti-NTM medical treatment and 22 received anti-fungal treatment. Furthermore, 1 case was lost to follow-up, 1 died from multiple organ failure, 7 were effectively cured of both TM and NTM), 9 relapsed, and 6 had persistent infection. Of the 13 patients with positive AIGA, only 1 patient (P17) received AIGA treatment. Upon receiving combined methylprednisolone and rituximab treatment, the AIGA titer of P17 decreased from more than 1: 10,000 to 1: 5000 after 2 courses of therapy. The total treatment time, including anti-fungal and anti-NTM, was 40 months (6–114 months) (Table 5).

**Table 5:** Treatment and patient outcomes in Group 1.

Patient	AIGAs	TM therapy	NTM therapy	AIGAs treatment	Duration	Outcome*
P1	Positive	VCZ + AMB 2w. Secondary prophylaxis VCZ 12 m	RFP + EMB + MXFX + CLR 6 m, then relapse after 1 m of withdrawal, changed to Biapenem + LVFX for 5 m	None	12 m	TM effective; NTM relapse
P2	Positive	Intravenous VCZ for 2w then oral VCZ 5 m	EMB + INH + RFP	None	15 m	TM relapse; NTM persistent infection
P3	Positive	Intravenous VCZ for 2w then oral VCZ for 6 m	LVFX + EMB	None	12 m	TM relapse; NTM persistent infection
P4	Positive	AMB for 2w, then oral VCZ 12 m	CLR + MXFX + RZA + SMZ for 7 m	None	20 m	TM and NTM effective
P5	Positive	Oral ICZ for 24 m	MXFX + EMB for 36 m	None	36 m	TM and NTM effective
P6	Positive	AMB for 2w, secondary prophylaxis oral ICZ for 4 m	CLR + MXFX for 6 m then relapse, change to MXFX + IMP for 6 m	None	12 m	TM and NTM relapse
P7	Positive	Oral ICZ for 12 m	INH + RFP + EMB + PZA	None	36 m	TM effective; NTM persistent infection
P8	Positive	AMB for 2w, then oral ICZ	CLR + AMK 7 m	None	43 m	TM and NTM effective
P9	Positive	Intravenous VCZ for 3 days then oral VCZ	None	None	3 days	Death
P10	Positive	Oral ICZ	None	None	60 m	TM and NTM persistent infection
P11	Positive	AMB for 2w, then oral ICZ	None	None	6 m	Lost to follow-up
P12	Positive	AMB for 2w, then oral ICZ for 18 m. VCZ for 60 m for relapse	CLR + MXFX	None	78 m	TM and NTM both relapse
P13	Positive	AMB for 2w, then oral VCZ	CLR + CXT + MXFX for 12 m; then AMK + IMP + AZM for 6 m for relapse	None	18 m	TM effective; NTM relapse
P14	Positive	ICZ for 12w	IMP + CLR for 36 m	None	40 m	TM and NTM Effective
P15^13^	NA	Micafungin	AZM + RFP + EMB	None	–	TM and NTM effective
P16^14^	Positive	AMB	RIF + EMB + CLR + CIP then relapse, changed to CIP + INH + RIF + CLR	None	60 m	TM and NTM persistent infection
P17^15^	NA	AMB for 5 m, then oral ICZ for 25 m	RFP + EMB + CLR for 19 m	None	41 m	TM and NTM effective
P18^16^	Positive	LAMB for 2w, then oral ICZ for 6 m	IMP + AMK for 1 m, then AMK + CLR + CIP for 3 m then relapse, changed to CLR + EMB for 1 m	Rituximab plus methylprednisolone	55 m	TM Effective; NTM relapse
P19^17^	Positive	AMB + ICZ for 2w, then oral ICZ	LXFX	None	–	TM and NTM effective
P10^18^	Positive	ICZ for 10 m	INH + RFP + PZA + EMB + MXFX for 24 m then relapse, and changed to INH + RFP + PZA + EMB + CLR + SMZ 6 m	None	69 m	TM effective, NTM relapse
P21^19^	Positive	ICZ	IMP for 6 m then relapse, then changed to MEM + AMK + TGC	None	78 m	TM effective; NTM persistent infection
P22^19^	Positive	AMB for 2w, then oral ICZ for 10w	INH + EMB + CLR + AMK + OFLX for 22 m, then changed to EMB + CLR + AMK + OFLX	None	114 m	TM effective; NTM relapse

*NA* anti-IFN-γ autoantibodies not detected, *AMB* amphotericin B, *LAMB* amphotericin B liposome, *VCZ* voriconazole, *ICZ* itraconazole, *EMB* ethambutol, *RFP* rifampin, *CIP* ciprofloxacin, *INH* isoniazid, *PZA* pyrazinamide, *OFLX* ofloxacin, *CXT* cefoxitin, *IMP* imipenem, *AMK* amikacin, *CLR* clarithromycin, *LXFX* levofloxacin, *MXFX* moxifloxacin, *SMZ* sulfamethoxazole, *MEM* meropenem, *TGC* tigecycline, *AIGAs* anti–interferon-γ autoantibodies.

*For 14 patients the outcome assessment was performed at their last outpatient follow-up. For 8 patients that were part of the systematic literature review, their outcome assessment was extracted from the literature. For the following patients, the duration between the time the treatment was stopped, and the outcome assessment was respectively: 12 months for P15; 19 months for P16; 10 months for P18; 12 months for P19; and 6 months for P22. For the following patients, the outcome assessment time was performed when they were discharged: P17, P21, and P21.

## Discussion

To our knowledge, this is the first report showing the differences between TM and NTM co-infection and their respective mono-infections. Some clinical differences were noticed across groups. The severity of inflammation (WBC, N, ESR, CRP), inflammatory anemia, and prevalence of involved sites in TM and NTM co-infection were more evident than in TM or NTM mono-infection, especially when compared. Noteworthy, when patients received single active antifungal or single anti-NTM treatment, some symptoms improved while others worsened. Inflammatory markers (WBC, N, CRP, ESR) did not significantly decline or increase, but did not maintain normal levels, indicating the presence of double or multiple infections, especially in patients with high-titer AIGAs.

Univariate analysis for risk factors of TM and NTM co-infection found that high level of AIGAs, WBC, N, HGB, IgG, IgM, IgA, serum globulin, ESR, and CRP and low level of CD4^+^T cells were taken as potential risk factors for TM and NTM co-infection. Most importantly, the titer of AIGAs was significantly positively correlated with the number of sites involved, which suggested that the titer of AIGAs was associated with disseminated infection. Thus, high-titer AIGAs may represent a potential risk factor and susceptibility factor for co-infection of TM and NTM in HIV-negative hosts. Monitoring the AIGA titer is the most important step in screening for co-infections or disseminated infections.

IFN-γ is produced principally by T lymphocytes and natural killer cells after stimulation with microbial products and interleukin (IL)-12^20^. Patients with positive AIGAs often suffer from recurrent infections, especially due to NTM^8,9,11^. Because IFN-γ is an activator of macrophage differentiation and a pro-inflammatory activator of innate immunity, the blockade effects of the AIGAs on IFN-γ present in the serum of patients with NTM are hypothesized to regulate the antimicrobial function of macrophages^20^. Recently, a study showed that AIGAs can neutralize IFN-γ, affect the activation of the IFN-γ receptor (IFN-γR), and downregulate the production of its downstream factors, such as TNF-α and IL-12, and inhibit IFN γ-STAT-1 phosphorylation^11^. IFN-γ is also an essential activator of CD4^+^T cell differentiation into Th1 cells^21^. In the present study, the AIGA titers and positive rates of patients with co-infection were significantly higher than those of other groups, while their CD4^+^T and CD3^+^T cell levels were significantly lower than those of other groups. Meanwhile, there was a significant negative correlation between AIGA titers and the number of CD4^+^T cells. Thus, the neutralizing and blockade effects of the AIGAs may be related to the low level of CD4^+^T cells, which may be the reason for patients susceptible to opportunistic pathogens, especially intracellular pathogens.

TM and NTM showed very similar clinical manifestations such as fever, anemia, weight loss, cough, expectoration, and skin lesions. They both can involve skin lesions, respiratory system, and bone, leading to local or disseminated infections. High recurrence and/or persistent infection rates (59.1%) was found in TM and NTM co-infected patients, primarily due to misdiagnosis and/or missed diagnoses as each other or TB. In HIV-negative individuals with TM and NTM co-infection, only one pathogen (TM or NTM) was discovered in the early stages of disease in most patients (77.3%). Moreover, inflammatory markers in TM and NTM co-infection were higher than in NTM mono-infection, though no significant difference was found between simultaneous and successive TM and NTM. These suggest that most patients found to have sequential TM and NTM infections were in fact infected with both TM and NTM simultaneously; however, one pathogen was missed at diagnosis, resulting in poor prognosis.

Furthermore, TM histopathology often manifests as granuloma, but caseous granuloma is rare, which was characteristic of positive TM cultures in this study. Second, it is more difficult to make a differential diagnosis of NTM from TB because of its similar histopathology and acid-fast staining. Thus, even if it is positive for acid-fast staining, metagenomic next-generation sequencing and culture of mycobacteria is essential to detect NTM, especially when anti-tuberculosis treatment is not effective. Third, TM and NTM co-infection has a higher inflammatory index and dissemination than NTM infection, which may be related to AIGAs and TM. Fourth, when a single treatment (anti-fungal or anti-tuberculosis branch) is not effective for a patient, potential co-infection with other pathogens should be considered, especially in patients with positive AIGAs.

## Conclusion

High-titer AIGAs represent an independent risk factor for TM and NTM co-infection in HIV-negative hosts. AIGA may be a major susceptibility factor for intracellular pathogens such as TM and NTM. Further, poor prognosis of TM and NTM co-infection may be due to misdiagnosis and/or missed diagnoses. Therefore, AIGA screenings in patients with unexplained recurrent or multiple microbial infections may serve as an indicator of acquired immunodeficiency.

## Limitations

There are important limitations to our study. First, the number of participants and reports was small, reflecting that AIGA disease and co-infection of TM and NTM is still rarely recognized. Second, it is unclear when the AIGA is positively detected or activated by infection. Despite these limitations, this is the first comprehensive description of TM and NTM co-infection in AIGA-associated immunodeficiency syndrome.

## Methods

## Study design and patients

### Guangxi, China cohort

For this multicenter, observational, retrospective cohort study, we screened for TM and NTM co-infection (Group 1) in HIV-negative patients from 13 hospitals between January 1st, 2012, and January 1st, 2020. Group 2 comprised patients with TM mono-infections and Group 3 of NTM mono-infections. All patients were HIV-negative. The healthy controls were recruited after completing a multicenter retrospective study and a systematic literature review. Healthy control volunteers (Group 4) were enrolled to match the gender, age, and HIV-negative condition of Group 1. Demographic and clinical data were recorded on standardized forms.

The 13 participating centers included: (1) The Eighth Affiliated Hospital of Sun Yat-Sen University; (2) The First Affiliated Hospital of Guangxi Medical University; (3) The Affiliated Tumor Hospital of Guangxi Medical University; (4) The Second Affiliated Hospital of Guangxi Medical University; (5) The Hospital of Guangxi Zhuang Autonomous Region; (6) Nan Xishan Hospital of Guangxi Zhuang Autonomous Region; (7) Nanning Second People's Hospital; (8) Nanning Forth People's Hospital; (9) Nanning Eighth People's Hospital; (10) Yiyang Central Hospital; (11) Liuzhou First People's Hospital; (12) Guigang First People's Hospital; and (13) Guilin First People's Hospital.

This study was approved by the Ethical Review Committee of the First Affiliated Hospital of Guangxi Medical University (2018.KY-E-094). The clinical trial was registered on www.clinicaltrials.gov (NCT03819348). Written informed consent was provided by all healthy participants in this study. All methods were performed in accordance with the relevant guidelines.

### Systematic literature review cohort

For a systematic review of articles related to TM/NTM co-infection, original articles published in English from Jan 2004 to July 2019 were reviewed using the following electronic databases: PubMed, Web of Science, Embase, and BIOSIS. Screening of relevant studies was based on combinations of keywords, such as “non-tuberculosis”, “non-tuberculous”, “non tuberculosis”, “nontuberculous”, “nontuberculous mycobacterium”, “nontuberculosis mycobacteria”, “NTM”, “MOTT”, “atypical mycobacterium”, “*penicilliosis*”, “*Penicillium marneffei”, “Talaromycosis*”, “*Talaromyces marneffei*”, “*T. marneffei”,* and “*P. marneffei”.* Inclusion criteria for the systematic literature review consisted of the following: (1) TM and NTM diagnosis based on exact pathogen, isolated NTM, and TM from clinical specimens; (2) articles clearly stating the HIV infection status; and (3) only HIV-uninfected subjects with TM and NTM co-infection were included. Informed consent was waived for patients in the literature review due to the nature of the study.

The data presented in this study result from a merge of these 2 cohorts (Guangxi cohort and literature review cohort). Clinical outcomes definitions: (1) Cured (no recurrence of TM and/or NTM infection for at least six months after discontinuation of antifungal/anti-NTM therapy); (2) persistent or relapsed infection (persistent infection: no improvement of clinical symptoms after antifungal/anti-NTM treatment, relapsed infection: improvement of clinical symptoms, negative pathogen detection after antifungal/anti-NTM effective treatment, followed by the reappearance of pathogen-associated infectious signs and/or positive pathogen testing); and (3) death. A disseminated disease was defined as an infection in at least two noncontiguous and sterile sites.

### Diagnostic criteria for NTM and TM

Each patient fulfilled the diagnostic criteria of each disease. NTM was diagnosed following the 2007 American Thoracic Society (ATS)/Infectious Disease Society of America guidelines^22,23^. TM infection was diagnosed as follows: (1) positive cultures for TM, characterized by dimorphic fungi that grew either as a mold at 25 °C or as yeast at 37 °C; (2) characteristic morphology of the yeast form of TM, confirmed by cytology and histopathology from tissues and secretions using Periodic Acid-Schiff (PAS) staining or Wright’s stain, including a transverse septum^23^; or (3) TM and/or NTM isolated by metagenomic next-generation sequencing from clinical specimens.

### Anti-IFN- γ autoantibody assay

Serum samples obtained under sterile conditions before the patient received antimicrobial therapy treatment and during the active stage of the infection. Serum samples were retrieved from a serum bank and stored at − 80 °C. AIGAs were detected in all participants. All serum samples were tested at the first thaw. The detection of AIGAs was performed using an enzyme-linked immunosorbent assay kit (Cloud-Clone Corp. Wuhan, China) whose detection range is 12–200 ng/ml. According to the manufacturer’s protocols: the serum samples from patients were 1:1500 diluted, and serum samples from a healthy control were 1:600 diluted by phosphate-buffered saline (PBS). The normal range for the anti–IFN-γ–autoantibody concentration was defined by the 99th percentile for the healthy controls and was estimated using the log-normal distribution. Outlying concentrations were classified as positive for anti–IFN-γ autoantibodies^1,6^.

### IFN-γ, IL-4, IL-6, IL-8, TNF-α assay

Serum samples obtained under sterile conditions before the patient received antimicrobial therapy treatment and during the active stage of the infection. Serum samples were retrieved from a serum bank and stored at − 80 °C. IFN-γ, IL-4, IL-6, IL-8, TNF-α were detected in all participants. All serum samples were tested at the first thaw. The detection of IFN-γ, IL-4, IL-6, IL-8, TNF-α was performed using a human enzyme-linked immunosorbent assay kit (Cloud-Clone Corp. Wuhan, China) according to the manufacturer’s instructions.

### Statistical analysis

Continuous variables were expressed as median ± interquartile range. Differences between groups were compared using Kruskal–Wallis H or Mann–Whitney U tests. Dunn-Bonferroni test was used for post-hoc comparisons. Chi-square or Fisher’s exact tests were used to compare categorical variables. Spearman’s correlation coefficient was used for ranked data to measure the dependence of two nonparametric variables. Univariate logistic analysis was used to estimate risk factors of co-infection. We used SPSS (version 25.0), and GraphPad Prism (version 7) for statistical analysis and graph illustrations, and a two-sided *P*-value of 0.05 or less was considered significant.

### Ethical approval

This study was approved by the Ethical Review Committee of the First Affiliated Hospital of Guangxi Medical University (2018.KY-E-094). The clinical trial was registered on www.clinicaltrials.gov (NCT03819348). Written informed consent was provided by all participants in the prospective cohort study.

### Consent to participate

All study participants provided informed consent, and the study design was approved by the appropriate ethics review board.

### Consent for publication

Written informed consent for publication was obtained from all participants.

## Supplementary Information

Supplementary Information.

## Data Availability

The datasets used or analyzed during the current study are available from the corresponding author on reasonable request.

## Code availability

We used SPSS (version 25.0), and GraphPad Prism (version 7) for statistical analysis and graph illustrating, and *P*-value < 0.05 was considered significant.
