# Supplementary material for: Talaromyces marneffei and nontuberculous mycobacteria co-infection in HIV-negative patients
Source: Sci Rep. 2021 Aug 10;11:16177. doi: 10.1038/s41598-021-95686-0 (PMC8355300; doi:10.1038/s41598-021-95686-0)
Supplement: Supplementary file 1 — Supplementary Information. [file 41598_2021_95686_MOESM1_ESM.docx]

Supplementary Table 1. **Laboratory** **findings in Group 1**

| Variable | | Group 1A(n=5) | Group 1B(n=17) | *P*-value |
| --- | --- | --- | --- | --- |
| WBC×10^9^cells/L | | 21.7(15.5, 23.7) | 22.3(18.4, 24.1) | 0.662 |
| N×10^9^ cells /L | | 17.0(12.7, 18.9) | 17.1(12.3, 18.7) | 1.000 |
| ESR mm/h | | 92(90, 108) | 106(91, 117) | 0.524 |
| CRP mg/L | 114(91.9, 172.1) | | 175.1(126.2, 196.0) | 0.298 |
| CD4+T cell cells/ul | | 258(124, 976) | 940(545, 1072) | 0.114 |
| CD8+T cell cell/ul | | 329(124, 1005) | 779(414, 886) | 0.181 |
| CD3+T cell cell/ul | | 765(280, 2092) | 1349(895, 1742) | 0.330 |

Group 1A: both TM and NTM pathogens were found simultaneously (simultaneous infection of TM and NTM), Group 1B: sequential infection status, including NTM infection before TM and TM infection before NTM. Abbreviations: IQR, interquartile range; WBC, white blood cell; RBC, red blood cell; N, Neutrophils; L, Lymphocyte; HGB, Haemoglobin; ESR, Erythrocyte sedimentation rate; CRP, C-reactive protein. Data are presented as the number of the median (IQR). Mann-Whitney U test was used to calculate *P*-values. *P* < 0.05.

**Supplementary Table 2. Inflammatory index after anti-fungal and anti-NTM treatment for TM and NTM in Group 1**

| **Variable** | **Before treatment** | **Single regimen therapy** | **Diagnosis Co-infection** | **After combined treatment** |
| --- | --- | --- | --- | --- |
| WBC×10^9^cells/L (Diagnosis TM before NTM) | 19.2(17.51, 22.06) | 11.07(8.56,13.32) | 24.55(15.74, 33.67) | 9.82(6.88, 11.00) |
| WBC×10^9^cells/L (Diagnosis NTM before TM) | 22.26(15.19, 24.51) | 6.99(4.49, 9.53) | 16.08(16.00, 37.5) | 8.56(4.12, 8.59) |
| WBC×10^9^cells/L (Diagnosis Co-infection) | 21.76(10.72, 23.58) | - | 22.01(20.28, 23.74) | 12.60(7.19, 13.83) |
| N×10^9^cells/L (Diagnosis TM before NTM) | 16.16(12.70, 18.85) | 5.98(2.76, 7.73) | 10.81(10.17, 16.06) | 6.88(3.60, 8.88) |
| N×10^9^cells/L (Diagnosis NTM before TM) | 18.21(13.06, 19.77) | 5.14(3.30, 8.56) | 7.40(5.00, 18.71) | 6.03(2.59, 6.66) |
| N×10^9^cells/L (Diagnosis Co-infection) | 17.02(12.70, 18.87) | - | 16.42(11.5, 17.9)- | 4.89(3.07,6.97) |
| ESR mm/h (Diagnosis TM before NTM) | 113(93.5, 129.5) | 76(87.0, 107.0) | 105.0(87.0, 107.0) | 36.0(36.0, 55.0) |
| ESR mm/h (Diagnosis NTM before TM) | 105.5(78.8, 142.8) | - | 95.5(34.0, 157.0) | 85.5(55.0, 116.0) |
| ESR mm/h (Diagnosis Co-infection) | 92.0 (90.0, 108.0) | - | 92.0 (90.0, 110.0) | 23.0(22.0, 26.0) |
| CRP mg/L (Diagnosis TM before NTM) | 192.00(164.02, 222.74) | 16.05(4.63, 195.56) | 192.00(124.62, 210.00) | 15.29(5.76, 29.15) |
| CRP mg/L (Diagnosis NTM before TM) | 126.20(38.77, 163.83) | 45.00(32.40, 64.44) | 138.00(119.10, 158.00) | 39.80(18.0, 56.63) |
| CRP mg/L (Diagnosis Co-infection) | 114.01(91.87, 172.09) | - | 93.17(72.34, 114.00) | 72.30(69.00, 78.00) |

Abbreviations: WBC, White blood cell; N, neutrophil counts; ESR, erythrocyte sedimentation rate; CRP, C reactive protein; TM, *Talaromyces marneffei*; NTM, nontuberculous mycobacteria.

Supplementary Table 3. Comparison of involvement sites among three groups

| Site | Group 1(n=22) | Group 2(n=22) | Group 3(n=22) | *P*-value |
| --- | --- | --- | --- | --- |
| Site |  |  |  | **0.007** |
| Lung | 17(77.3) **^b^** | 20(90.1) **^c^** | 22(100) | **0.015** |
| Lymph node | 20(90.1) **^b^** | 20(90.1) **^c^** | 7(31.8) | **0.000** |
| Skin | 19(86.4) **^b^** | 10(45.5) **^c^** | 2(9.1) | **0.000** |
| Bone/Joint/muscle | 12(54.5) **^b^** | 15(68.2) **^c^** | 1(4.5) | **0.000** |
| Pleural | 12(54.5) **^b^** | 18(81.8) **^c^** | 7(31.8) | **0.004** |
| Pericardium | 2(9.1) | 7(31.8) | 2(9.1) | 0.076 |
| Spleen/liver/peritoneum | 4(18.2) | 6(27.3) | 2(9.1) | 0.438 |
| Central nervous system | 0 | 2(9.1) | 0 | - |

**^a^** indicates statistical significance between Groups 1 and 2.

**^b^** indicates statistical significance between Groups 1 and 3.

**^c^** indicates statistical significance between Groups 2 and 3.

Data are presented as n (%). Fisher’s exact test and Kruskal-Wallis H test were used to calculate *P*-values. *P* < 0.05. Group 1 = patients with TM and NTM co-infection, Group 2 = patients with TM infection only, Group 3 = patients with NTM infection only.

Supplementary Table 4. Distribution of nontuberculous mycobacterial species and **other co-infection pathologies** in Group 1 patients

| Species and sites | Number of patients (n/%) N=22 |
| --- | --- |
| Species* |  |
| Rapid-growing | 7/11 (63.6) |
| Mycobacterium abscessus | 4/11 (36.4) |
| Mycobacterium chelonae | 3/11 (27.3) |
| Mycobacterium fortuitum | 1/11 (9.1) |
| Slow-growing | 6/11 (54.5) |
| Mycobacterium kansasii | 3/11 (27.6) |
| Mycobacterium avium complex | 1/11 (9.1) |
| Mycobacterium intermedium | 1/11 (9.1) |
| Mycobacterium avium-M | 1/ (9.1) |
| Unidentified | 11 |
| co-infected other pathologies† | 9 (40.9) |
| Staphylococcus aureus | 3 (13.6) |
| Aspergillus | 3 (13.6) |
| Salmonella | 3 (13.6) |
| Burkholderia | 2 (9.1) |
| Albicans Saccharomyces | 1 (4.5) |
| Stenotrophomonas maltophilia | 1 (4.5) |
| Mycobacterium tuberculosis | 1 (4.5) |
| Klebsiella pneumoniae | 1 (4.5) |
| Providenciarettgeri | 1 (4.5) |
| Tuberculosis | 1 (4.5) |
| varicella zoster virus | 1 (4.5) |
| herpes simplex virus | 1 (4.5) |

Data are presented as n (%). *Among these 22 patients, two were infected with two subtypes of NTM: *Mycobacterium marinum* and *Mycobacterium sphaeroides*; and *Mycobacterium avium* and *Mycobacterium fortuitum*. † In nine patients co-infected with other pathologies, one patient could be infected with up to six pathogens during the course of the disease.
